# Lead Induced Ototoxicity and Neurotoxicity in Adult Guinea Pig

**DOI:** 10.1155/2019/3626032

**Published:** 2019-01-15

**Authors:** Yanni Zhang, Qian Jiang, Shaobing Xie, Xuewen Wu, Jun Zhou, Hong Sun

**Affiliations:** ^1^Department of Otolaryngology Head and Neck Surgery, Xiangya Hospital of Central South University, Changsha, Hunan 410008, China; ^2^Province Key Laboratory of Otolaryngology Critical Diseases, Changsha, Hunan 410008, China; ^3^Department of Central Laboratory, Xiangya Hospital of Central South University, Changsha, Hunan 410008, China

## Abstract

Lead exposure causes or aggravates hearing damage to human or animal, but the detailed effects of lead exposure on auditory system including injury sites of the cochlea in mammal remain controversy. To investigate the effect of chronic lead exposure on auditory system, 40 adult guinea pigs with normal hearing were randomly divided into five groups. They were fed 2 mmol/L lead acetate in drinking water for 0, 15, 30, 60, and 90 days (n = 8), respectively. Lead concentrations in blood, cochlea, and brainstem were measured. Auditory function was measured by auditory brainstem response (ABR) and distortion product otoacoustic emission (DPOAE). The morphology of cochlea and brainstem was observed, and expression of autophagy-related protein in brainstem was also assessed. The blood lead concentration reached a high level at the 15th day and kept stable, but the lead level in brainstem and cochlear tissue increased obviously at the 60th day and 90th day of lead exposure, respectively. There was no significant difference in the morphology of hair cells and stria vascularis (SV) among these five groups, but the number of spiral ganglion neuron (SGN) gradually decreased after 60 days. The differences of ABR thresholds and DPOAE amplitudes were not statistically significant among each group, but I wave latency, III latency, and I-III wave interval of ABR were delayed with the prolonging of time of lead exposure. The expressions of autophagy-related protein ATG5, ATG6, and LC3B in brainstem were increased after 30 days. These results suggest that the key target of lead toxicity was the auditory nerve conduction pathway including SGNs and brainstem, rather than cochlear hair cells and SV. Autophagy may play a very important role in lead toxicity to auditory nervous system.

## 1. Introduction

With the development of modern industry, environmental pollution has become a common public concern in China, and heavy metal like lead pollution has captured the medical profession's attention. Lead accumulation in the body can lead to irreversible dysfunction in multiple systems [1–3]. Many studies have shown that lead exposure can cause or aggravate hearing damage in human or animals [4–6]. Lead exposure experiments in C57BL/6 mice by Samson Jamesdanie proved that lead exposure could damage the inner ear barrier, activate the oxidative stress pathway in the mouse cochlea, and significantly alter hearing threshold [7]. Jones LG et al. found that lead exposure during development could change the structure of axons and brainstem auditory nuclei [8]. Liu X et al. [4] testified that lead exposure for 8 weeks caused morphological changes of cochlear outer hair cells of rats and guinea pigs and induced rupture and missing of tight junctions between endothelial cells and border cells on the SV. Based on our previous in vitro study, we demonstrated that lead could induce damage on cochlear spiral ganglions (SGNs) and their nerve fibers after 72 hours of treatment at 2 mM, but no obvious detrimental effects on nearly all cochlear hair cells [9]. In addition, some other studies also have confirmed that lead had no significant effect on cochlear outer hair cells and ABR during lead exposure [10], and Jones LG et al. also found that short-term lead exposure might be the contribution of demyelization of auditory nerve fibers in the midbrain of mice but no significant change in ABR threshold [8]. Therefore, the detailed effects of lead exposure on auditory system including injury sites of the cochlea in mammal remain controversy. It is also not clear whether the autophagy signaling pathway plays a role in the auditory system toxicity caused by lead. This study was designed to observe the changes of cochlear hair cells, SV, SGNs, and brainstem of guinea pigs after chronic lead exposure.

## 2. Materials and Methods

### 2.1. Animals and Lead Exposure

40 normal adult guinea pigs (200- 220 g) of both sexes were included in this study. All animals were tested for Preyer reflex and checked for physical appearance before the experiment to insure they were healthy and normal. Then they were randomly divided into five groups (A, B, C, D, and E) with eight animals in each group. All animals were purchased from the animal experimental center of the Xiangya Medicine School, Central South University. All animals were kept in the same raising environment (SPF level) and were fed with pure water containing 2 mmol/L lead acetate (Hunan university chemical decontamination agent factory, Kaifu district, Changsha city, Hunan province) for 0, 15, 30, 60, and 90 days, respectively. All procedures regarding the use and care of animals in this study conformed to the NIH guidelines and were approved by the Institutional Animal Care and Use Committees of the Central South University.

### 2.2. Auditory Function Evaluation

Auditory functions of the guinea pigs in all groups were evaluated by examination of ABR and DPOAE after feeding with lead containing water for 0, 15, 30, 60, and 90 days, respectively. The animals were anesthetized with 10% chloral hydrate (Central South University Xiangya Hospital). The TDT RZ6 hearing test system and the TDT BioSigRP software were used to detect ABR and DPOAE in the screen room.

#### 2.2.1. ABR

Click was used as the sound stimulation, and the speaker was about 2 cm away from the testing ear. The sound intensity of the speaker was calibrated by an acoustic calibrator. When testing the ABR, the reference electrode in the silver needle electrode was inserted under the skin of the same side of the ear, the recording electrode was inserted under the mesial top of the skull, and the ground electrode was inserted under the skin of the contralateral ear. The ABR waveform viewing window was 10 ms, the amplitude was amplified 20 times by the amplifier, and the band-pass filter was 100-3000 Hz. 512 stimulus presentations delivered at 21/s were averaged to obtain the waveform of ABR. Replications were executed at stimulus levels near or at threshold. The lowest stimulus level that elicited a repeatable 2-phase waveform was considered to be the ABR threshold for the testing ear.

#### 2.2.2. DPOAE

For measuring DPOAE, earplugs connecting the two sound emitters were inserted into the external auditory canal of the guinea pig. When the connection was stable, we closed the electro-acoustic shielding room, ran the BioSigRZ software, and retrieved the preset DPOAE sound file. We did the same procedure on the guinea pig's other ear and repeat the above steps after saving the measured waveform.

### 2.3. Determination of Lead Concentration in Blood, Cochlea, and Brainstem

Anesthetized with 10% chloral hydrate (Central South University Xiangya Hospital), 2 ml of heart blood of guinea pigs was taken out from the atrium, and then heart perfusion was performed with 0.9% physiological saline until the blood was nearly colorless, and then the guinea pig was quickly decapitated. After the intact brainstem tissue removed, the temporal bone was quickly taken away, and then the bulla was opened to expose the cochlea. The cochlear tissue containing basement membrane and the spiral ligament in one cochlea of each guinea pig was dissected immediately under the microscope. The specific method was detailed presented in our previous publication [11]. The other cochlea was prepared for morphological observation. Brainstem tissues, cochlear basement membranes, and spiral ligament tissues were collected and placed on a circular filter paper to dry. The tissues were placed into 1.5 ml EP tube after weighing, then adding 0.6 ml of concentrated nitric acid (65%-68%). The solution was clear when dissolved for 7 days and then diluted with 1 ml concentrated nitric acid. The blood, Brainstem, and cochlear specimens were sent to the Pediatric Laboratory of Xiangya Hospital of Central South University to measure the lead concentration by Bohui (BH2101S) atomic absorption spectrometer.

### 2.4. Morphological Observation of Cochlear Hair Cells, SV, and SGNs in Cochlea

The other cochlea of each guinea pig was prepared for morphological observation. The temporal bones were quickly removed. The apex of the cochlea, the round window, and the oval window were punctured. Fixation was performed with 10% formalin in PBS at 4°C perfusing through the cochlear apex and then the samples were immersed in the fix solution for 24 hours. Then the basilar membranes of some cochleae were microdissected out as described in our previous publications [9]. The cochlear basilar membranes were stained with Alexa488-labeled phalloidin (Thermo Fisher Scientific, Cat. No. A12379) for 1 hour and followed by 3 times rinse with PBS. After staining, they were placed on glass slides covered with glycerin and cover slip and examined under a confocal laser scanning microscope (Leica TCS SP8) with appropriate filter to detect green carboxyfluorescein of Alexa488-labeled phalloidin. Images were captured and analyzed with a Leica Image Examiner and processed with Adobe Photoshop. Some cochleae were decalcified by 10% EDTA for 14 days. After that, the cochleae were embedded in paraffin according to the conventional procedure. Finally, HE staining was performed. The specific steps were consistent with those described in the literature [12]. Under the 400-fold magnified field of the microscope (Leica DMI4000B), we observed the morphology of SV cells, and the criteria for a neuron to be counted were a well-defined cytoplasm and nuclear membrane, as well as clear nucleoli within the nucleus. “Double-counting” was not a consideration as the nucleolus was small as it was compared with section thickness and every one in six series was chosen for cell counting ensuring that there were 60 *μ*m between analyzed sections [13]. In this way an estimated neuronal number for each sample was obtained for each animal.

### 2.5. Morphological Observation of Brainstem

Rapid decapitation followed anesthetizing guinea pigs with 10% chloral hydrate (Xiangya Hospital of Central South University). Part of the brainstem tissue was fixed in formalin solution for 24 hours, and the other part was stored frozen in a -80°C refrigerator for autophagy-related proteins detection. The brainstem tissue in formalin was fixed to be embedded, sliced, and stained with HE according to the conventional paraffin section method, and the morphology and quantity of the cells were observed under an optical microscope (Leica DMI4000B).

### 2.6. Expression of Autophagy-Related Proteins in Brainstem

The dissected and homogenized brainstem was placed in a lysis buffer containing 20 mM Tris-HCl (pH7.5), 150 mM NaCl, 1 mM Na2EDTA, 1mMEGTA, 1% Triton, 2.5 mM sodium pyrophosphate, 1 mM *β*-glycerophosphate, and 1 mM Na3VO4 (Cell Signaling Technology, Beverly, MA). Additional constituents were added as follows with their final concentration of 0.5% Na-deoxycholate, 0.5% sodium dodecyl sulfate (SDS), 1 *μ*M okadaic acid, 1 mM phenylmethylsulfonyl fluoride (PMSF), 0.1 mg/ml benzamidine, 8 *μ*g/ml calpain inhibitors I and II, and 1 *μ*g/ml of each leupeptin, pepstatin A, and aprotinin. Homogenates were sonicated for 30 seconds before centrifugation at 50,000 rpm for 20 minutes. Supernatants were assayed for protein concentration (Bio-Rad Protein Assay; Bio-Rad, Hercules, CA), and aliquots were stored at -80°C for subsequent use. Autophagy-associated proteins ATG5, ATG6, and LC3B were detected by Western blotting; namely, proteins were separated by SDS-polyacrylamide gel electrophoresis (PAGE) by using 4–12% gradient polyacrylamide gels (Invitrogen, Carlsbad, CA) and then transferred to PVDF membranes (Immobilon-P, Millipore, Bedford, MA) followed by immunoblotting, and antibodies were purchased from Novus Biologicals (NB110-53818, NBP1-00085, NB100-2220).

### 2.7. Data Analysis

All statistical analysis was performed with GraphPad Prism5 software. Student t tests were applied to compare the differences among groups, and P<0.05 was considered to indicate a statistically significant difference.

## 3. Results

### 3.1. Lead Concentration in Blood, Cochlear Tissue, and Brainstem

**Figure 1:**
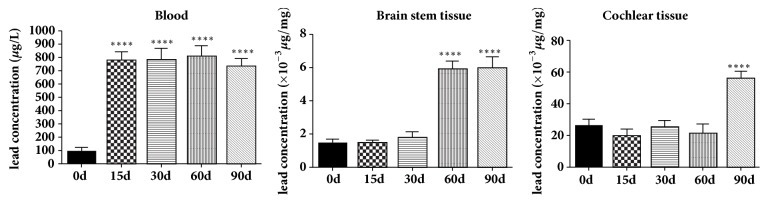
Lead concentrations of adult guinea pigs of groups A-E. As lead exposure time is prolonged, blood lead levels are raised and stabilized from day 15 forward, brainstem tissue lead concentration is raised and stabilized from 60th day on, and the lead concentration in the cochlear tissue began to rise at day 90.

As lead exposure time going on, the blood lead levels of adult guinea pigs increased gradually and reached a stably high level at the 15th day (Figure 1). The lead concentrations in blood of A-E groups were 89.50 ± 16.78, 772.5 ± 32.51, 775.8 ± 44.54, 799.3 ± 42.95, 726.5 ± 31.42 *μ*g/L, respectively (n = 8). The lead concentrations in brainstem tissue increased significantly at the 60th day and then remained stable (Figure 1), and those for A-E groups were 1.45 ± 0.1, 1.46 ± 0.08, 1.79 ± 0.17, 5.88 ± 0.26, 5.92 ± 0.36 (*μ*g/g), respectively (n = 8). Meanwhile the lead concentrations in cochlear tissues of A-E group were 26.1 ± 2.19, 19.63 ± 2.16, 25.28 ± 2.18, 21.48 ± 2.95, 56.13 ± 2.24 (*μ*g/g), respectively (n = 8) (Figure 1).

### 3.2. Pathological Changes of Cochlear Hair Cells after Lead Exposure

**Figure 2:**
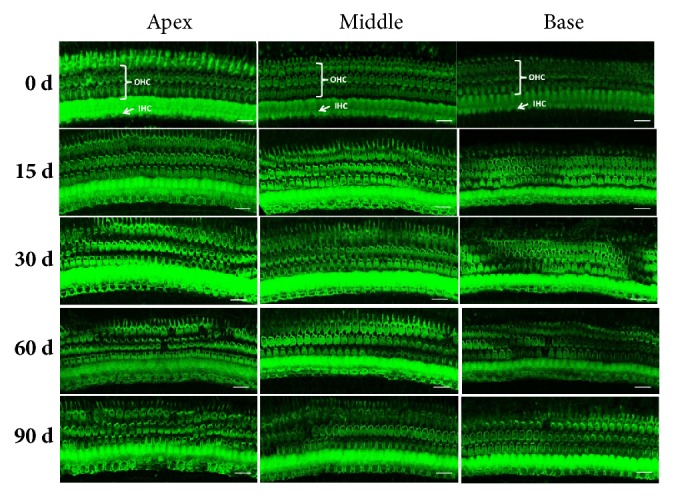
Representative confocal photomicrographs of normal control and lead exposure groups. The OHCs and IHCs were stained with Alexa 488-labeled phalloidin (green). The number and shape of three rows of OHC and one row of IHC appeared normal in the basilar membrane from apical turn to basilar turn in 0 d, 15 d, and 30 d group. Only few OHC appeared missing in 60 d and 90 d group. OHC: out hair cells; IHC: inner hair cells. Scale bar: 20 *μ*m.

The morphology of the inner hair cells and most outer hair cells of the cochlea of each group remained normal although lead exposure time was increasing. Their location, array, number, and shape did not change significantly, the cilia arranged neatly, and there was no obvious dislodging or other damage appeared under microscopy, only few out hair cells missing at the 60th day and 90th day (Figure 2).

### 3.3. Pathological Changes of Stria Vascularis Cells after Lead Exposure

**Figure 3:**
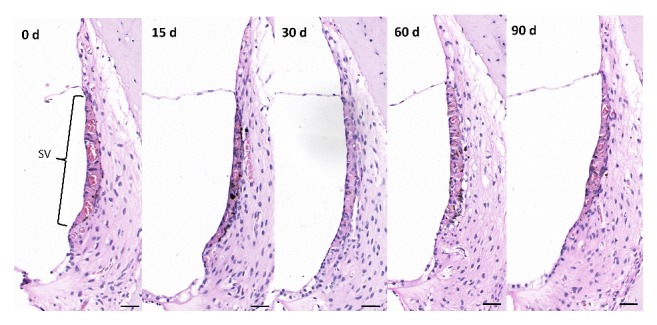
HE staining of SV in the cochlea. The number and shape of SV cells were normal in each group. SV: stria vascularis. Scale bar: 100 *μ*m.

During the lead exposure time, the stria vascularis cells of the cochlea of each group arranged regularly, and the number and shape did not change significantly (Figure 3).

### 3.4. Morphological Changes of Spiral Ganglion Cells in the Cochlea

**Figure 4:**
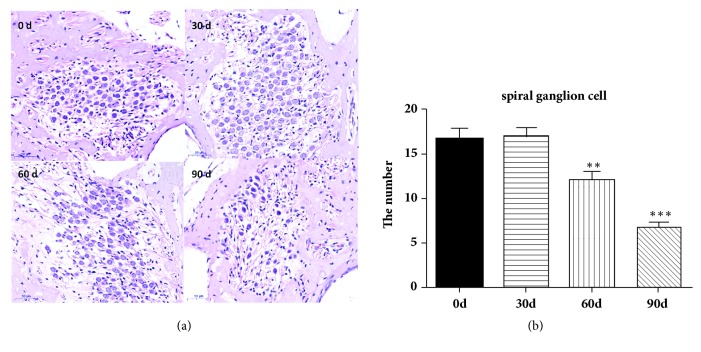
Morphological observation of SGNs in the cochlea. As lead exposure time was prolonged, the number of SGNs gradually decreased, cell filaments were broken, and some cells exhibited nuclear fragmentation and karyopyknosis and others apoptosis phenomena from 60 days forward (a). The number of SGNs at 60 and 90 days was statistically different from that before lead exposure (b). Scale bar: 50 *μ*m.

On day of 60 after lead exposure, the number of SGNs in the cochlea began to decrease, cell filaments were broken, and some cells exhibited nuclear fragmentation and karyopyknosis and others apoptosis phenomena. At the 90th day, most cells disappeared, and only a small amount of SGNs remained (Figure 4(a)). The numbers of SGNs at 60 days and 90 days were statistically different from that at 0 days, respectively (p < 0.01 and p < 0.001) (Figure 4(b)).

### 3.5. ABR and DPOAE Results

**Figure 5:**
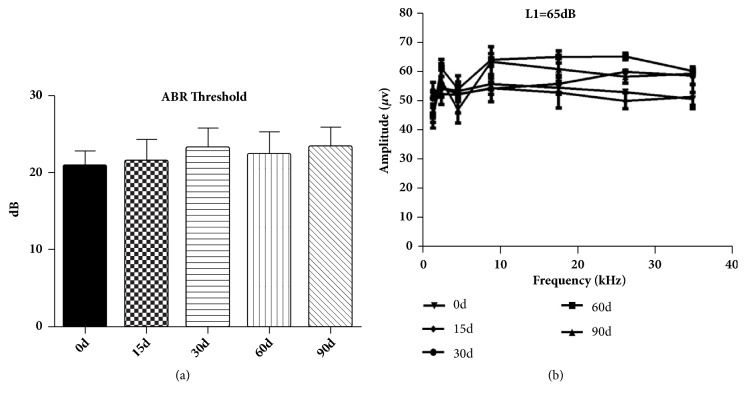
ABR threshold of adult guinea pigs in A-E group. With the increase of lead exposure time, there was no significant change in the ABR threshold between each group (a), and there was also no significant difference in DPOAE among groups (b).

The average ABR thresholds of A-E group induced by click were 20.83 ± 0.83, 21.67 ± 1.05, 23.33 ± 1.05, 22.50 ± 1.12, and 23.33 ± 1.05 dB SPL, respectively (n = 8). There was no significant difference among groups (p>0.05) (Figure 5(a)). The DPOAE test demonstrated no significant change in the amplitude of DPOAE at all frequencies at days 0, 15, 30, 60, and 90 (Figure 5(b)).

**Figure 6:**
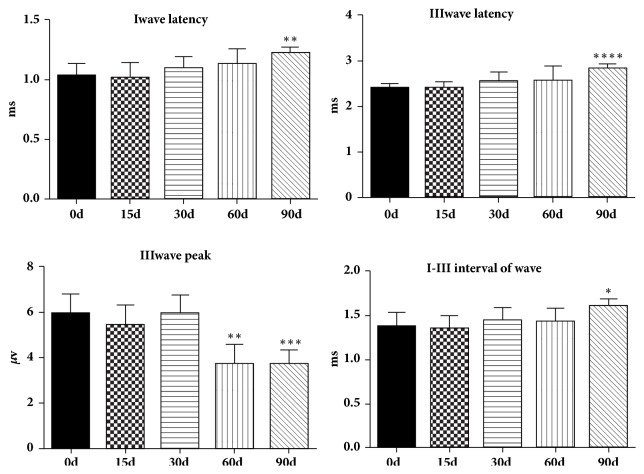
Changes of latency and amplitude of ABR in adult guinea pigs between groups A-E. With the increase of lead exposure time, the latency of I and III waves and the interval of I-III waves were prolonged, and the peak of III waves began to decrease from the 30th day forward.

At the level of 80 dB SPL click sound stimulation, the latencies of I wave in A-E group of adult guinea pigs were 1.03 ± 0.04, 1.01 ± 0.05, 1.10 ± 0.03, 1.13 ± 0.06, and 1.22 ± 0.02 ms, respectively (n = 8) (Figure 6, Table 1); there was a statistically significant difference between group A and group E (p<0.01), but there was no statistically significant difference between the rest groups with group A (p> 0.05). The latencies of III wave in A-E group were 2.41 ± 0.04, 2.37 ± 0.06, 2.54 ± 0.08, 2.56 ± 0.13, and 2.83 ± 0.04 ms, respectively (n = 8) (Figure 6); the III wave latency from group A to group E increased gradually, with statistical significance between group E and group A (p<0.01), but the difference was not statistically significant among the rest groups and group A (p>0.05). The peaks of III wave in group A-E were 5.94 ± 0.35, 5.43 ± 0.37, 5.99 ± 0.31, 3.77 ± 0.33, and 3.75 ± 0.23 *μ*V, respectively (n = 8) (Figure 6); comparing with group A, the peaks of III wave in both group D and E deceased significantly (p<0.001). The intervals of I-III wave were 1.38 ± 0.07, 1.36 ± 0.06, 1.45 ± 0.06, 1.44 ± 0.06, and 1.61 ± 0.03 ms, respectively (n = 8) (Figure 6); there was a statistically significant difference between group A and group E (p<0.01), but there was no statistically significant difference among the rest groups and group A (p> 0.05).

### 3.6. Expression of Autophagy-Related Proteins in Brainstem

**Figure 7:**
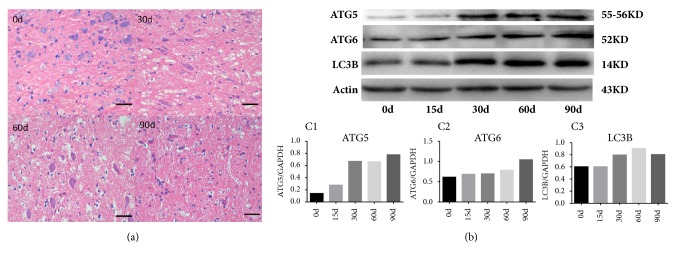
Morphological changes of brainstem tissue (a) and the expression level of autophagy-related proteins (b) in each group. Gray value analysis of autophagy-related proteins (C1-C3). There was no obvious damage in brainstem tissue morphology (a), but the protein level of ATG5, ATG6, and LC3B increased significantly from 30 days to 90 days of lead exposure (b, c). Scale bar: 100 *μ*m.

With the increase of lead exposure time, there was no obvious damage to brainstem tissue morphology (Figure 7(a)). However, the expression level of autophagy-related protein ATG5, ATG6, and LC3B increased significantly from 30 days to 90 days (Figure 7(b)).

## 4. Discussion

Lead is an important neurotoxic agent in the environment, and it can especially cause irreversible central nervous system damage during children's development [2, 14]. Recent studies have shown that even low level of lead exposure can cause hearing impairment in adults and children [15, 16]. Choi conducted a correlation analysis of blood lead and the hearing level on 3698 adults in the United States between 1999 and 2004 from national health and nutrition survey, which showed the highest level of lead exposure group compared with the lowest level lead exposure group; the hearing threshold increased 18.6%, suggesting that lead exposure is an important contribution for hearing loss [16].

This study proved that, after continuous lead exposure, the lead concentration in the blood of adult guinea pigs increased rapidly and stabilized at a higher level from the day of 15. However, the lead concentration in brainstem tissue increased significantly from the 60th day forward and kept stable and that in cochlear tissue did not increased significantly until at the 90th day of lead exposure. Lead is a heavy metal element that cannot be degraded in the environment and has an accumulation effect to body. The time difference of lead accumulation in different tissues may be due to the role of the blood-brain barrier and the blood labyrinth barrier. The blood exchange in the auditory system was abundant. Under the continuous stimulation of blood containing high concentration of lead, damage in tissues will appear eventually. Our result indicated that the auditory system began to show signs of toxic injury after 30 days of lead exposure; therefore, the intervention to prevent hearing loss should be executed in time. Although some studies suggested that the cochlear damage caused by lead poisoning was an important cause of hearing loss, the exact location in the hearing system still remains controversy. Liu X et al. [4] insisted that lead exposure for 8 weeks could trigger loss of cochlear outer hair cells and damage of tight junctions between endothelial cells and border cells on the stria vascularis. However, other authors believed that the main auditory damage caused by lead toxicity was in the auditory nervous system rather than in the hair cells. Buchanan LH et al. examined blood lead and DPOAE of 53 children (102 ears) aged 6-16 years and found no significant differences in DPOAE amplitude between lead exposure children and control children, as well as blood lead levels and DPOAE amplitude. No correlation between DPOAE signal and noise ratio (S/N) suggesting that lead exposure had no effect on cochlear outer hair cells [17]. Liang et al. applied 0.1-100 *μ*mol/L lead to in vitro cultured cochlear outer hair cells and found that the shape of the outer hair cells remained normal, and only outward potassium current slightly changed, which indicated that lead acetate has no significant effect on electrokinetic properties of the outer cochlear hair cells [18]. Similarly, in our previous and the present studies, we found that the cochlear hair cells remained intact regardless of the lead exposure time both in vitro and in vivo, intact functions of cochlear hair cells in the hearing test were further confirmed by DPOAE monitoring, and no obvious damage was found in SV as well. As the lead exposure time increased, the cochlear damage appeared and mainly manifested in the cell body and nerve fibers of the SGNs, indicating that the lead damage to the auditory system was confined in cochlear neural-system. Our study also found that although lead exposure had no significant effect on ABR threshold, the conduction amplitude of the auditory pathway from the cochlea to the midbrain tended to decrease with lead exposure time increasing. All the evidences from our experiments showed that the lead toxicity was mainly manifested in the auditory nerve conduction pathway.

Autophagy is a process of phagocytosis through which its own cytoplasmic protein or organelles were engulfed and coated into vesicles, then fusion with lysosomes forms autophagic lysosomes, in which the cell contents were degraded and recycled, and cells are able to maintain normal function and satisfy its own metabolic needs. Autophagy is highly dependent on the availability of the autophagy-related proteins (ATGs). Along with others, ATG5 is recognized as a key player in the formation of autophagosomes, which may also have potential key contribution in apoptosis [19–21]. Our experiment demonstrated that autophagy-related proteins ATG5, ATG6, and LC3B in brainstem tissue were significantly increased after 30 days of lead exposure, suggesting the autophagy activity in brainstem after 30 days of lead exposure. These proteins are also closely related to apoptosis, suggesting that the neurotoxicity by lead exposure may be related to apoptosis. Studies have shown that autophagy can lead to premature aging of auditory cells. An experiment carried out by Tsuchihashi, NA et al. has shown that when HEI-OC1 auditory cells were incubated with 5 mM H_2_O_2_ for inducing senescence, autophagy-related protein expressions were increased after one-hour H_2_O_2_ treatment, and after six hours, ATG7 and LC3-II protein expressions peaked. Transmission electron microscopy found lipofuscin and aggregates in autologous lysosomes, which are accumulated significantly in the cytoplasm of HEI-OC1 cells 48 hours after H_2_O_2_ treatment [22]. Giordano, S et al. demonstrated that autophagy was involved in the process of injury repair in the nervous system [23]. Although the expression of autophagy-associated protein in brainstem tissue increased significantly after 30 days of lead exposure, the damage of SGNs occurred and the corresponding hearing impairment appeared at 60 days of lead exposure as well, indicating that autophagy may play a protective effect in the early stage of lead exposure. In conclusion, there is a possibility that autophagy plays a very important role in lead ototoxicity and neurotoxicity in auditory nervous system.

## 5. Conclusion

This study suggested that the target of lead toxicity was mainly in the auditory nerve conduction pathway including SGNs and brainstem, rather than in cochlear hair cells and SV. Autophagy may play a very important role in lead ototoxicity and neurotoxicity in auditory nervous system. The intervention to prevent hearing loss caused by lead poisoning should be executed in time.

## Figures and Tables

**Table 1:** I, III wave latency, III wave peak, and I-III wave interval of ABR in each group.

group	80 dB SPL			
	I wave latency (ms)	III wave latency (ms)	III wave peak (*µ*V)	I-III wave interval (ms)
A	1.03 ± 0.04	2.41 ± 0.04	5.94 ± 0.35	1.38 ± 0.07
B	1.01 ± 0.05	2.37 ± 0.06	5.43 ± 0.37	1.36 ± 0.06
C	1.10 ± 0.03	2.54 ± 0.08	5.99 ± 0.31	1.45 ± 0.06
D	1.13 ± 0.06	2.56 ± 0.13	3.77 ± 0.33*∗*	1.44± 0.06
E	1.22 ± 0.02*∗*	2.83 ± 0.04*∗*	3.75 ± 0.23*∗*	1.61 ± 0.03*∗*

Note: *∗*: comparing with group A, all the I, III wave latency, III wave peak, and I-III wave interval of group E and III wave peak of group D had statistical significance (p<0.001); there was no significant difference among the rest groups.

## Data Availability

The image data used to support the findings of this study are included within the article.

## References

[1] Li N., Liu X., Zhang P. (2015). The effects of early life lead exposure on the expression of interleukin (IL) 1*β*, IL-6, and glial fibrillary acidic protein in the hippocampus of mouse pups. *Human and Experimental Toxicology*.

[2] Flores‐Montoya M. G., Sobin C. (2015). Early chronic lead exposure reduces exploratory activity in young C57BL/6J mice. *Journal of Applied Toxicology*.

[3] Cabarkapa A., Borozan S., Živković L. (2015). Implications of oxidative stress in occupational exposure to lead on a cellular level. *Toxicological and Environmental Chemistry*.

[4] Liu X., Zheng G., Wu Y. (2013). Lead exposure results in hearing loss and disruption of the cochlear blood-labyrinth barrier and the protective role of iron supplement. *NeuroToxicology*.

[5] Liu S., Zhang K., Wu S. (2011). Lead-induced hearing loss in rats and the protective effect of copper. *Biological Trace Element Research*.

[6] Wildemann T. M., Weber L. P., Siciliano S. D. (2015). Combined exposure to lead, inorganic mercury and methylmercury shows deviation from additivity for cardiovascular toxicity in rats. *Journal of Applied Toxicology*.

[7] Jamesdaniel S., Rosati R., Westrick J., Ruden D. M. (2018). Chronic lead exposure induces cochlear oxidative stress and potentiates noise-induced hearing loss. *Toxicology Letters*.

[8] Jones L. G., Prins J., Park S., Walton J. P., Luebke A. E., Lurie D. I. (2008). Lead exposure during development results in increased neurofilament phosphorylation, neuritic beading, and temporal processing deficits within the murine auditory brainstem. *Journal of Comparative Neurology*.

[9] Xue–wen W., Da–lian D., Hong S., Hong L., Hai–yan J., Salvi R. (2011). Lead neurotoxicity in rat cochlear organotypic cultures. *Journal of Otology*.

[10] Yamamura K., Terayama K., Yamamoto N., Kohyama A., Kishi R. (1989). Effects of acute lead acetate exposure on adult guinea pigs: electrophysiological study of the inner ear. *Fundamental and Applied Toxicology*.

[11] Wu X., Ding D., Jiang H. (2012). Transfection using hydroxyapatite nanoparticles in the inner ear via an intact round window membrane in chinchilla. *Journal of Nanoparticle Research*.

[12] Zheng G., Zhu Z., Zhu K., Wei J., Jing Y., Duan M. (2013). Therapeutic effect of adeno-associated virus (AAV)-mediated ADNF-9 expression on cochlea of kanamycin-deafened guinea pigs. *Acta Oto-Laryngologica*.

[13] Zhao J., Lurie D. I. (2004). Cochlear ablation in mice lacking SHP-1 results in an extended period of cell death of anteroventral cochlear nucleus neurons. *Hearing Research*.

[14] Mazumdar M., Bellinger D. C., Gregas M., Abanilla K., Bacic J., Needleman H. L. (2011). Low-level environmental lead exposure in childhood and adult intellectual function: a follow-up study. *Environmental Health: A Global Access Science Source*.

[15] Rooney J. P. K., Dórea J. G. (2012). Hearing loss in US adolescents and exposure to heavy metals: mercury in perspective. *Archives of Otolaryngology - Head and Neck Surgery*.

[16] Choi Y.-H., Hu H., Mukherjee B., Miller J., Park S. K. (2012). Environmental cadmium and lead exposures and hearing loss in U.S. adults: The National Health and Nutrition Examination Survey, 1999 to 2004. *Environmental Health Perspectives*.

[17] Buchanan L. H., Counter S. A., Ortega F. (2011). Environmental Lead Exposure and Otoacoustic Emissions in Andean Children. *Journal of Toxicology and Environmental Health, Part A. Current Issues*.

[18] Liang G. H., Jarlebark L., Ulfendahl M., Bian J. T., Moore E. J. (2004). Lead (Pb2+) modulation of potassium currents of guinea pig outer hair cells. *Neurotoxicology and Teratology*.

[19] Liu H., He Z., Simon H. (2015). Protective role of autophagy and autophagy-related protein 5 in early tumorigenesis. *Journal of Molecular Medicine*.

[20] Le Bars R., Marion J., Le Borgne R., Satiat-Jeunemaitre B., Bianchi M. W. (2014). ATG5 defines a phagophore domain connected to the endoplasmic reticulum during autophagosome formation in plants. *Nature Communications*.

[21] Otomo C., Metlagel Z., Takaesu G., Otomo T. (2013). Structure of the human ATG12~ATG5 conjugate required for LC3 lipidation in autophagy. *Nature Structural & Molecular Biology*.

[22] Tsuchihashi N. A., Hayashi K., Dan K. (2015). Autophagy through 4EBP1 and AMPK regulates oxidative stress-induced premature senescence in auditory cells. *Oncotarget*.

[23] Giordano S., Darley-Usmar V., Zhang J. (2014). Autophagy as an essential cellular antioxidant pathway in neurodegenerative disease. *Redox Biology*.

